# Are Surface Electromyography Parameters Indicative of Post-Activation Potentiation/Post-Activation Performance Enhancement, in Terms of Twitch Potentiation and Voluntary Performance? A Systematic Review

**DOI:** 10.3390/jfmk9020106

**Published:** 2024-06-17

**Authors:** Philip Gallardo, Giannis Giakas, Giorgos K. Sakkas, Panagiotis V. Tsaklis

**Affiliations:** 1 Department of Physical Education and Sport Science, ErgoMech-Lab, University of Thessaly, 421 00 Trikala, Greece; pgallardo@uth.gr (P.G.); ggiakas@gmail.com (G.G.); gksakkas@gmail.com (G.K.S.); 2 Department Molecular Medicine and Surgery, Growth and Metabolism, Karolinska Institute, 171 77 Solna, Sweden; 3 Center of Orthopaedics and Regenerative Medicine (C.O.RE.)/(C.I.R.I.), Aristotle University Thessaloniki, 541 24 Thessaloniki, Greece

**Keywords:** post-activation potentiation, electromyography, conditioning activities, post-activation performance enhancement

## Abstract

The aim was to identify if surface electromyography (sEMG) parameters are indicative of post-activation potentiation (PAP)/post-activation performance enhancement (PAPE), in terms of twitch potentiation and voluntary performance. Three databases were used in April 2024, with the following inclusion criteria: (a) original research, assessed in healthy human adults, and (b) sEMG parameters were measured. The exclusion criteria were (a) studies with no PAP/PAPE protocol and (b) non-randomized control trials. The following data were extracted: study characteristics/demographics, PAP/PAPE protocols, sEMG parameters, twitch/performance outcomes, and study findings. A modified physiotherapy evidence database (PEDro) scale was used for quality assessment. Fifteen randomized controlled trials (RCTs), with a total of 199 subjects, were included. The M-wave amplitude (combined with a twitch torque outcome) was shown to generally be indicative of PAP. The sEMG amplitudes (in some muscles) were found to be indicative of PAPE during ballistic movements, while a small decrease in the MdF (in certain muscles) was shown to reflect PAPE. Changes in the H_max_/M_max_ ratio were found to contribute (temporally) to PAP, while the H-reflex amplitude was shown to be neither indicative of PAP nor PAPE. This review provides preliminary findings suggesting that certain sEMG parameters could be indicative of PAP/PAPE. However, due to limited studies, future research is warranted.

## 1. Introduction

It is well documented that warming up prior to any physical activity may reduce the risk of musculoskeletal injuries [1,2] and can produce a noticeable improvement in athletic performance [3,4]. One of the mechanisms that has been of interest to power-strength athletes and coaches is the muscle potentiation effect that has been observed following warmups [5]. This enhancement, in response to voluntary muscle contractions, has been coined post-activation potentiation (PAP) and refers to a state where the contractile properties of the skeletal muscle(s) are acutely enhanced following a brief high-intensity contraction [5,6,7]. The PAP phenomenon was originally confirmed by measuring the maximum twitch force (or peak twitch torque [PTT]) that is evoked by supramaximal electrical stimulation [6,8], with PTT and the maximal twitch rate of torque development (RTD_TW_) being the most common twitch outcomes of PAP, also referred to as twitch potentiation [6,9,10]. Compared to post-tetanic potentiation, which is induced involuntary via high-frequency electrical tetanic stimulation, PAP refers to the enhanced twitch response following voluntary muscle contraction (at maximal or near-maximum intensities) [6,8]. Numerous mechanisms have been proposed to cause this potentiated twitch response or PAP effect, but currently it is believed that the primary mechanism for PAP is an increased expression of myosin regulatory light chain (MRLC) phosphorylation [5,11]. During maximal or near-maximum voluntary contractions, an increased influx of sarcoplasmic calcium (Ca^2+^) into the myoplasm upregulates the expression of skeletal muscle myosin light-chain kinases (skMLCKs) [11,12]. This increased expression of skMLCK phosphorylates the myosin subfragment-1 (S1) head closer to its joints with the subfragment-2 (S2) portion [12,13]. This sequentially augments the probability of a cross bridge to occur, improving the myosin head’s mobility, and allows potentiated fibers to improve their rate of force development (RFD), and this consequently enhances its contractility [5,11,12].

Practically, many sports coaches and athletes have therefore employed several different PAP strategies or conditioning activity (CA) protocols, with the aim of acutely enhancing voluntary force production and overall athletic performance [6,14]. Among the athletic population, various CA protocols have been demonstrated to be effective for acutely enhancing voluntary muscular performance, including athletes involved in rugby [10,15], track and field [10], soccer [16], volleyball [17], and football [10]. However, a major limitation in the PAP literature is that the term has loosely been used to explain all acute improvements in voluntary performance, following different CA protocols, without a direct twitch verification test (i.e., observing an acute increase in PTT or RTD_TW_) [6,9,10]. As accumulating evidence indicates that acute improvements in voluntary performance can occur independent of any changes in PAP (by its classical definition), the term ‘post activation performance enhancement’ (PAPE) has in more recent years been used to describe the acute improvements in voluntary muscular performance following different CAs [6,18]. Common approaches to evaluate PAPE usually include observing acute improvements in plyometric performance, such as countermovement jump (CMJ) height [15,18] and squat jump (SJ) height [16,18], in addition to enhanced sprinting speed [19,20], RFD, and peak power output (PPO) in different movements [16,18], following different CAs.

Further, while various CA protocols have been demonstrated to acutely enhance voluntary muscular performance, some interventional studies have actually reported a reduction in performance following different CA protocols [21,22], which has primarily been attributed to fatigue. Although there is no standard definition of fatigue [23], at the neuromuscular level, it has been defined as the failure to sustain a specified force output with a muscle or muscle group during exercise [24,25]. Numerous research instruments have been employed to distinguish between intramuscular (peripheral) and central (neural) processes that contributes to fatigue within the neuromuscular system, such as electrical stimulation [26], the interpolated twitch technique [27], and electromyography (EMG)/surface electromyography (sEMG) [28,29]. Among these research tools, sEMG has commonly been used as a convenient non-invasive tool to assess neuromuscular fatigue [29]. In particular, EMG spectral variables (e.g., mean power frequency [MPF] and median power frequency [MdF]) and the sEMG amplitude (e.g., the mean absolute value [MAV] and root mean square [RMS]) have commonly been evaluated [30,31,32], as changes usually occur in these parameters during fatiguing muscular activity [30,32,33]. Although sEMG has extensively been used to indirectly estimate neuromuscular fatigue [31], there is conflicting findings regarding the use of different sEMG parameters for assessing neural mechanisms that could modulate the PAP/PAPE response [34,35].

Normally, muscle potentiation and fatigue coexist when performing any muscular activity [36]. However, the post-stimulus state will be affected by the net balance between these two factors. Depending on the dissipation of fatigue and the decay rate of potentiation following recovery, there may be a net potentiated effect, a net attenuated effect, or an unaltered state in comparison to the pre-stimulus state [5,36]. Further, experimental evidence suggests that the net balances between the PAP/PAPE response and neuromuscular fatigue are also influenced by training experience [37], the rest period [38], and the intensity of the CA [39], making the use of sEMG to provide insights into the neural mechanisms of PAP/PAPE more complex. Interestingly, assessing changes in PTT and muscle compound action potentials or M-waves (evoked via electrical stimulation and with the use of various sEMG channels) has in recent years been used as a method for confirming the presence of PAP [10,40,41]. The M-wave has commonly been used to examine peripheral properties of the neuromuscular system without the involvement of the central nervous system [42,43]. Further, importantly, eliciting the maximal M-wave (M_max_), via supramaximal nerve stimulation, activates all motor units of the pool, including the fast-twitch units [44], which are more responsive to MRLC phosphorylation (i.e., the primary mechanism of PAP) [6,45]. By assessing the M-wave, researchers can control for changes in neuromuscular propagation that could influence the PTT in the stimulated muscle (e.g., changes in sarcolemmal membrane excitability [10,46,47]. Furthermore, another common parameter elicited with electrical stimulation, and assessed with sEMG, is an electrically induced spinal reflex or H (Hoffmann)-reflex [48]. The H-reflex measures the potency of synaptic transmission [48,49] and is frequently used to denote α-motoneurons’ excitability [49,50,51]. Still, our understanding of the H-reflex and its contribution to PAP/PAPE following different CAs’ protocols is currently limited, especially the ratio between the maximum H-wave (H_max_) amplitude and the M_max_ amplitude (i.e., the EMG H_max_/M_max_ ratio) [48], which is an index of the excitability of the motoneuron pool [44,48]. While accumulating data suggest that there may be different mechanisms that contribute to a potent PAP and PAPE response [6,8], data regarding the neural contribution of both PAP and PAPE are still limited [52,53].

Additionally, since there are several parameters of sEMG, such as the EMG amplitude (e.g., MAV and RMS), EMG spectral variables (e.g., MPF and MdF), H-reflex parameters (e.g., amplitude and threshold [54]), and M-wave values (e.g., amplitude, area, and duration [55]), it is unclear if some parameters of sEMG could provide better or worse insights to the neural mechanisms that could modulate the PAP/PAPE response. Having a greater understanding of how sEMG parameters relate to PAP/PAPE may help us reduce the incidence of musculoskeletal injuries in athletes and the general population, by providing us with new tools to construct better warmup guidelines. Clinically, this may also be of great importance for healthcare professionals rehabilitating patients with muscle weakness, a condition affecting millions of older adults worldwide [56]. Hence, the aim of this systemic review was to identify if sEMG parameters are indicative of PAP/PAPE, in terms of twitch potentiation and voluntary performance. Since an increase in sEMG amplitude in most instances is proportional with increasing muscle force [30] and increased MPF and MdF has been found to be indicative of a higher proportion of type II fiber recruitment [57,58], two hypotheses were proposed: (1) the PAP/PAPE magnitude will be positively related to the sEMG amplitude of the working muscle group (s), and (2) short-term increases in the sEMG frequency variables of the working muscle group (s) will also be positively related to PAP/PAPE.

## 2. Materials and Methods

### 2.1. Information Sources and Search Strategy

This systematic review was carried out following the PRISMA (Preferred Reporting Items for Systematic reviews and Meta-analyses) statement protocol [59] and was registered in INPLASY under the registration number INPLASY202460047. The literature search was performed until April 2024 in three relevant electronic databases: PubMed, Web of Science, and SCOPUS. The following keywords, in combination with Boolean operators (AND, OR) were used: “post activation potentiation”, “post activation performance enhancement” and “electromyography”. The advanced search in PubMed was used, with the following search entered in the query box: “electromyography” [All Fields] OR “EMG” [All Fields] OR “H-reflex” [All Fields] OR “M-wave*” [All Fields]) AND “post activation p*” [All Fields]. For Web of Science, the advanced search query builder was used, with the following search: ((((ALL = (“electromyography”)) OR ALL = (“EMG”)) OR ALL = (“H-reflex”)) OR ALL = (“M-wave*”)) AND ALL= (“postactivation potentiation”). Lastly, the advanced search with all fields in SCOPUS was used, with the following search: {electromyography} OR {EMG} OR {H-reflex} OR {M-wave} AND {post activation potentiation}. The results were filtered by article/document type in each database (Clinical Trial in PubMed, Article in Web of Science, and Article in SCOPUS, respectively). All studies were saved on the Zotero (6.0.37) bibliographic reference manager [60].

### 2.2. Inclusion/Exclusion Criteria

The following inclusion criteria were applied: (a) the study was an original research article, and the PAP/PAPE protocols were conducted in healthy human adults (i.e., no recent illness and no injuries), and (b) sEMG recordings were an outcome variable of interest. The exclusion criteria were (a) studies with no explicit PAP/PAPE protocol, and (b) interventional studies with no control group or counterbalance.

### 2.3. Data Extraction

For all included articles, the following information was extracted: (a) study characteristics (author, year, and sample size); (b) subjects’ demographics (sex, age, and training status); (c) CA protocols (type of exercise and load); (d) sEMG parameters; (e) twitch/performance measures (PAP and PAPE outcomes, respectively); (f) study findings.

### 2.4. Methodological Quality Evaluation

The modified physiotherapy evidence database (PEDro) scale was used to evaluate the methodological quality of the included studies in the review (Table 1), all of which were randomized control trials (RCTs). Items 5–7 were removed from the original PEDro scale, as blinding the subjects and investigators in supervised exercise interventions is not always feasible. This has been conducted in previous systematic reviews of exercise interventions [57,58]. Accordingly, this modified PEDro scale consisted of 8 items, and included eligibility criteria (item 1), randomization (item 2), concealed allocation (item 3), the groups being similar at baseline (item 4), more than 85% retention (item 8), intention-to-treat analysis (item 9), between-group comparison (item 10), and point measures and measures of variability (item 11). The highest score on this modified rating scale was 7, as the first item was not counted in the total score. The methodological quality was categorized as follows: poor quality (≤3 points), moderate quality (4 points), good quality (5 points), and excellent quality (6–7 points).

## 3. Results

### 3.1. Study Selection and Results of Literature Retrieval

**Figure 1:**
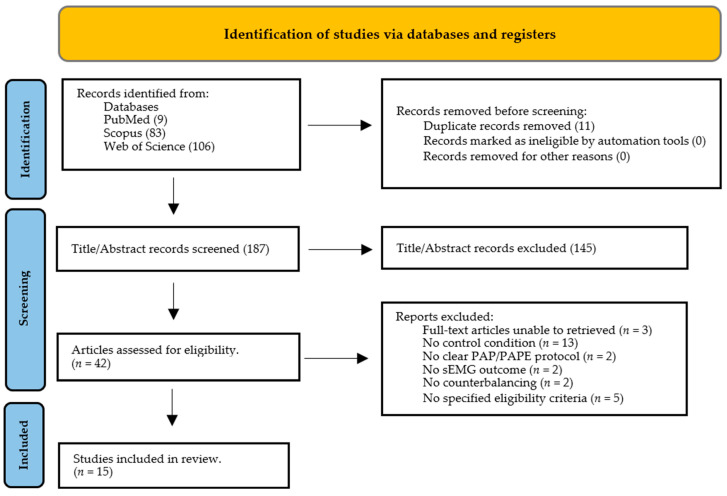
Flow diagram of screening process.

The search of PubMed, Web of Science, and SCOPUS databases provided a total of 198 records. After deduplication, 187 records remained (Figure 1) and 145 records were excluded based on their titles and abstracts. After reviewing the full text, fifteen underwent full data extraction and were included in the review (Table S1). Among the included studies, twelve examined the sEMG amplitude, six the M-wave, two the MdF, two the H-reflex, and one the EMG H_max_/M_max_ ratio as sEMG parameters. The five most common twitch/performance measures to detect PAP/PAPE among the included studies were PTT, PPO, RFD, and CMJ height. The flow diagram is presented in Figure 1, and it illustrates the screening process and the main reasons for exclusion. 

### 3.2. Characteristics of Studies Included

All studies included in this review were RCTs and had a cross-over design [10,15,16,17,18,34,35,43,48,61,62,63,64,65,66]. In total, 11 of 15 RCTs used separate two-way repeated-measures analyses of variances (ANOVAs) to evaluate the interaction between the time and different CA protocols on sEMG and PAP/PAPE outcomes, respectively [10,15,16,17,18,34,35,43,48,61,63]. Additionally, three RCTs used separate multivariate analyses of variance (MANOVAs) to determine the influence of different CA protocols on several sEMG and PAP/PAPE outcomes, respectively [18,65,66], while three other RCTs used one-way repeated measures ANOVAs to compare the effect of time on different sEMG and PAP/PAPE outcomes, respectively [43,62,63]. The sample size in the studies varied from 8 [48] to 20 [15,43], with a total of 199 subjects included in this review. Fourteen of the included studies used low (i.e., 30% to 40% of 1 repetition maximum [1RM] [16,64]), moderate (i.e., 60% to 70% of 1RM [61,64] or ∼70% of maximized mechanical-power output [P_max_] [17]), and high intensity (i.e., ≥80% of 1RM or ≥100% of P_max_, to isometric maximum voluntary contraction [iMVC]) resistance exercise as a CA [9,17,18,34,35,48,61,62,63,65,66]. The most common combinations of sEMG parameters and PAP/PAPE outcomes included sEMG amplitude and PPO [15,16,17,18,61], M-wave and PTT [35,48,62,63], and sEMG amplitude and CMJ height [15,16,18]. The remaining two studies [10,43] used a drop jump (DJ) protocol as a CA, with one study examining PTT and the M-wave [10], and another investigating repeated sprint ability (RSA) outcomes and RMS amplitude, M-wave, and MdF, respectively [43]. Table 2 provides a full description of the included studies.

### 3.3. Quality of the Studies (Risk of Bias)

Results of the PEDro rating are presented in Table 1. The fifteen RCTs in this review had an average score of 6.07 (mean = 6.07 ± 1.27) and were therefore deemed as having “excellent” methodological quality (on average). Still, most studies had methodological deficits concerning “concealed allocation” (14 of 15 studies). Moreover, five studies were discarded after assessing the quality, due to not having specified eligibility criteria (Figure 1), and consequently were deemed as having a high risk of bias.

### 3.4. Synthesis of Results

Changes in sEMG parameters and twitch/performance outcomes between different CA protocols were examined. The evidence was summarized for conditions with sEMG parameters indicative of acute improvements in twitch/performance outcomes (PAP and PAPE, respectively) in healthy athletic adults, but also incidents where they were deemed as unrelated.

#### 3.4.1. The sEMG Amplitude (MAV and RMS) and PAP/PAPE

Twelve studies examined the sEMG amplitude and the occurrence of PAP/PAPE [15,16,17,18,34,35,43,61,63,64,65,66]. All twelve studies demonstrated that the PAP/PAPE outcomes increased (*p* < 0.05), while there were no significant changes in the sEMG amplitude (in some muscles) (*p* > 0.05). However, noteworthily, eight studies also reported that both the sEMG amplitude (in different muscles) and certain PAP/PAPE outcomes (in particular PAPE) increased significantly [15,16,17,18,34,43,64,66] after performing either a series of DJs [42] or low- to high-intensity resistance exercise [15,16,17,18,34,64,66] as a CA (*p* < 0.05).

##### The MAV and PAP/PAPE

One RCT in this review used the MAV to detect the sEMG amplitude, and in addition assessed the effect of individualized intra-complex recovery intervals (ICRIs) [15]. In this study, Scott and colleagues found that the sEMG amplitude increased (in several lower body muscles) following either a hex bar deadlift (HBD) or BS-CA protocol, respectively, with moderate intensity (i.e., 70% of 1RM) and an additional 23% accommodating resistance, compared to baseline. Specifically, an increase in the VL (20.4%, *p* < 0.01), BF (22.7%, *p* < 0.01), tibialis anterior (TA) (22.0%, *p* < 0.01), and GM (21.9%, *p* < 0.01) sEMG amplitude was noted, together with acute improvements in PPO (4.0%, *p* < 0.01) and CMJ height (8.5%, *p* < 0.01), respectively, when the ICRIs were individualized. Notably, these short-term increases in sEMG amplitude, PPO, and CMJ height disappeared when ICRIs were instead fixed and prescribed at either 30 s, 90 s, or 180 s following the HBD and BS protocol (*p* > 0.05).

##### The RMS Amplitude and PAP/PAPE

Eleven studies examined the RMS amplitude and the occurrence of PAP/PAPE. One high-quality RCT [16] reported that four sets of low-intensity back squats (BSs) with either 50%, 60%, or 70% of arterial occlusion pressure (AOP), during blood flow restriction training (BFRT), were all effective for increasing the RMS amplitude in the vastus medialis (VM) (10.8% to 15.2%, *p* < 0.05), vastus lateralis (VL) (5.6% to 9.5%, *p* < 0.05), rectus femoris (RF) (5.9% to 8.3%, *p* < 0.05), and biceps femoris (BF) (9.6% to 25%, *p* < 0.05), compared to the control (BS with no AOP).

The highest RMS values were observed in the 70% AOP condition for all the muscles, followed by the 60% AOP condition. However, an acute increase in CMJ and SJ performance (i.e., PPO, RFD, and vertical jump height, respectively) was only noted in the 50% and 60% AOP groups (5–10 min post the BS-BFRT protocol). Further, the greatest improvements in the CMJ-PPO (6.3%, *p* < 0.05), SJ-PPO (3.2%, *p* < 0.05), CMJ-RFD (2.4%, *p* < 0.05), SJ-RFD (2.3%, *p* < 0.05), CMJ height (6.7%, *p* < 0.05), and SJ height (6.4%, *p* < 0.05) were observed in the 50% AOP condition compared to the control (5 min post the BS-BFRT protocol). Interestingly, however, the gluteus maximus (GM) RMS amplitude decreased significantly (−6.6% to −4.9%, *p* < 0.05) in all AOP groups compared to the first set (baseline), with the lowest and highest values observed in the 50% and 70% AOP groups, respectively. Contrarily, another high-quality RCT by Mina and colleagues [18] reported a short-term increase in the VL RMS amplitude (27.5% to 33.4%, *p* < 0.05), PPO (4.4% to 5.9%, *p* < 0.05), RFD (12.9% to 19.1%, *p* < 0.05), and CMJ height (5.3% to 6.5%, *p* < 0.05), 30 s to 12 min following a high-intensity BS protocol with variable resistance (VR), compared to pre-intervention values.

Noteworthily, the VL RMS amplitude was only significantly higher than the pre-intervention values when it was expressed as mean concentric RMS amplitude, as no significant differences were observed for peak concentric VL RMS amplitude (*p* > 0.05) nor mean eccentric VL RMS amplitude (*p* > 0.05). Although, interestingly, an earlier study by the same researchers [66] revealed that the mean eccentric RMS amplitude of the quadriceps femoris (QF) acutely increased (32.2%, *p* < 0.01) after performing a chain-loaded resistance (CLR) BS protocol (85% of 1RM, with 35% of the total load generated with chains), compared to a control (i.e., standard BS at 85% of 1RM). Of note, the same study found that the CLR-BS protocol acutely enhanced the subjects’ maximum strength (i.e., their 1RM load on the BS) (6.2%, *p* < 0.05), compared to the control. However, similarly, the QF sEMG activity was not significant when it was expressed as peak concentric QF RMS amplitude (*p* > 0.05), peak eccentric QF RMS amplitude (*p* > 0.05), nor mean concentric QF RMS amplitude (*p* > 0.05).

Contrariwise, nonetheless, another high-quality RCT [17] found that the mean concentric QF RMS amplitude increased (7.0% to 19.3%, *p* < 0.05), after performing a loaded SJ-CA protocol, with either moderate (i.e., 70% of P_max_) or high intensity (i.e., 130% of P_max_) compared to a control (no SJs). This higher QF activity was observed 1–10 min post the respective CA in the 130%P_max_ and 70%P_max_ trial. Intriguingly, short-term improvements in PPO (during repeated jumps) were only noted 5 min (13.9%, *p* < 0.05) and 7 min (8.2%, *p* < 0.05) post the CA in the 130%P_max_ and 70%P_max_ conditions, respectively, which coincided with the highest RMS values in each respective condition, compared to the control. Contrarily, two other high-quality RCTs [34,64] reported that the pectoralis major (PM) and triceps brachii (TB) RMS amplitude was only elevated when it was analyzed during the highest mean propulsive velocity (MVP) in the concentric phase of a bench press throw (BPT), although the intensity of the CA protocol, the muscle used for the EMG analysis, and accumulated neuromuscular fatigue was shown to influence how they were related. Specifically, one RCT [64] investigated the effect of different bench press (BP) velocities (performed as fast as possible until the mean velocity dropped to 90% [C90] vs. 70% [C70] of the fastest repetition) and different intensities (40% vs. 60% of 1 RM). In this study, the authors demonstrated that the TB RMS amplitude was only significantly higher (28.6%, *p* < 0.01) in the C90 condition (i.e., less accumulated neuromuscular fatigue) with 60% of 1 RM (i.e., moderate intensity), compared to baseline. In addition, this higher TB activity was accompanied with the greatest short-term improvements in the MVP (9.2%, *p* < 0.01) and PV (7.5%, *p* < 0.01), respectively, although no significant differences were observed for the PM RMS amplitude in this study (*p* > 0.05).

Contrariwise, in another study [34], the same authors observed that the PM RMS amplitude acutely increased (40% to 43.3%, *p* < 0.01), 8–12 min following a high intensity (i.e., 80% of 1RM) BP-C90 protocol, compared to baseline, and this coincided with the greatest acute improvements in the MVP (8.5%, *p* < 0.01) and PV (6.0%, *p* < 0.01), respectively. However, this time, no significant differences were observed for the TB RMS amplitude (*p* > 0.05). Moreover, one RCT by Zagatto and colleagues [43] used a DJ-CA protocol and assessed its effect on repeated sprint performance. In this study, a higher BF RMS amplitude (16.4%, *p* < 0.05) was reported, followed by significant improvements in RSA outcomes (mean time = −3.4%, *p* < 0.05; slowest time = −3.8%, *p* < 0.05; total time = −3.4%, *p* <0.05), compared to a control (no exercise condition).

Furthermore, two moderate- to good-quality RCTs [43,63] in this review reported that the sEMG amplitude actually decreased (in different lower body muscles), while a PAP/PAPE effect was observed. Specifically, the same study by Zagatto and colleagues [43] also noted that the RF RMS amplitude decreased during the 4th (−24.9%, *p* = 0.021) and 10th (−30.7%, *p* = 0.035) repeated sprint compared to the 1st sprint after the DJ protocol, while there were significant improvements in RSA outcomes. Contrarily, in the other study [63], the medial and lateral gastrocnemius (MG and LG, respectively), soleus (SOL) and TA muscle, and several performance outcomes were investigated following a 6-s plantar flexion iMVC. In this study, the MG RMS amplitude acutely decreased immediately following the iMVC (−20%, *p* < 0.05), compared to the control (no iMVC condition), and this surprisingly coincided with the greatest short-term improvements in PTT (178.6%, *p* < 0.05). Importantly, however, most studies in this review used different normalization procedures for the EMG signal. Nevertheless, the muscle of interest was most commonly normalized to the RMS values obtained during a 1 RM attempt [34,64] or squat isometric lifts [18,66].

#### 3.4.2. The MdF and PAP/PAPE

Two studies examined the MdF and the occurrence of PAP/PAPE (specifically PAPE outcomes) [16,43]. One RCT [43] demonstrated that the MdF increased in the VL after a DJ protocol (9.3%, *p* < 0.05), but no significant differences was observed compared to a sled towing trial or the control (no exercise) (*p* > 0.05). Still, an improvement in RSA outcomes was noted in the DJ condition, compared to the control (*p* < 0.05). Further, in another RCT [16], it was reported that four sets of BS with either 50%, 60%, or 70% AOP reduced the Mdf of the VM (−17.4% to −10.0%, *p* < 0.05), VL (−9.2% to −14.3%, *p* < 0.05), RF (−11.9% to −4.0%, *p* < 0.05), BF (−18.9% to −9.2%, *p* < 0.05), and Gmed (−5.8% to −4.2%, *p* < 0.05), respectively, compared to the control. The highest drop in MdF was observed in the 70% AOP condition in most muscles, followed by the 60% AOP condition. Still, short-term performance improvements were only observed in the 50% and 60% AOP conditions, respectively, with the greatest effects observed in the 50% AOP condition compared to the control (5 min post the BS-BFRT protocol). Additionally, the MdF of the GM only increased (7.8%, *p* < 0.05) in the 70% AOP condition. Moreover, all studies in this review [16,43] demonstrated that various PAPE outcomes significantly increased (*p* < 0.05), while there were no changes in the MdF in other muscles (*p* > 0.05).

#### 3.4.3. The M-Wave and PAP/PAPE

Six studies investigated the occurrence of PAP/PAPE and changes in the evoked M-wave response (peak-to-peak amplitude [PtpA]) obtained from either the VM [10,48], VL [35,43], soleus (SOL) [62], MG [63], or LG [63]. Five studies used motor nerve stimulation to obtain the M-wave [35,43,48,62,63], while one study in this review used direct muscle stimulation (from the VL) [10]. Nevertheless, all studies found that the M-wave PtpA remained unchanged, while different PAP/PAPE outcomes increased at certain time points (*p* < 0.05). Specifically, five RCTs [10,35,48,62,63] found that the M-wave remained constant while the PTT increased after performing a CA protocol with either isokinetic knee extensions (2.6% to 4.9%, *p* < 0.05) [35], plantar flexion iMVCs [62,63] (13.8% to 178.6%, *p* < 0.05), DJs [10] (15% to 23%, *p* < 0.001), or knee extension iMVC (6.7% to 66.6%, *p* < 0.05) [48], compared to either baseline values or a control group. This PAP response occurred immediately (2–10 s) after the CA [10,48,62,63] and remained significant for up to 30 s to 18 min [10,48,62,63] depending on the CA protocol.

Further, one study reported a significantly higher RTD_TW_ (32%, *p* < 0.001), with no changes in the M-wave (*p* > 0.05), immediately after a DJ-CA protocol [10], compared to a control (no DJs). In addition, three RCTs noted short-term improvements in either RSA performance outcomes (*p* < 0.05) [43] or voluntary peak torque (2.1% to 6.1%, *p* < 0.05) [35,63] compared to baseline, following a CA protocol with DJs [43], plantar flexion iMVCs [63], or isokinetic knee extensions [35]. However, one RCT [62] observed a short-term increase in the M-wave PtpA of the SOL, immediately after three series of 5 s plantar flexion iMVCs (and explosive plantar flexions), compared to baseline, with increases at the first iMVC (8.7%, *p* < 0.05), second iMVC (10.6%, *p* < 0.05), and 2–30 s post the last iMVC (5.5% to 12.2%, *p* < 0.05), although it rapidly dropped to non-significant values at 90 s post the last iMVC (*p* > 0.05). Noteworthily, the PTT in the SOL was significantly higher at the second iMVC (15.2%, *p* < 0.05) and 2–90 s post the last iMVC (13.8% to 18.8%, *p* < 0.05), but the values became non-significant at 150 s post the last iMVC (*p* > 0.05). Interestingly, a short-term improvement in plantar flexor isometric RFD (iRFD) was also noted when the RFD profile was analyzed in discrete units of time (25.0% to 31.6%, *p* < 0.05) at 15–60 s post the CAs, although no significant changes were observed when the iRFD was expressed as time to peak force (T_peak_) or average RFD (RFD_avg_) (*p* > 0.05).

#### 3.4.4. The H-Reflex and PAP/PAPE

Two studies evaluated the presence of the PAP/PAPE and changes in the evoked H-reflex response (PtpA) obtained from either the VM [48] or the SOL [62]. Both studies used motor nerve stimulation to obtain the H-reflex and found that it remained unchanged (*p* > 0.05), while different PAP/PAPE outcomes increased at certain time points (*p* < 0.05). In particular, Hodgson and colleagues [62] observed a significant increase in the PTT of the SOL (13.8–18.8%, *p* < 0.05) compared to baseline (2–90 s post iMVC), while the H-reflex remained constant. In the other study, Folland and colleagues [48] observed a significantly higher maximal twitch force (i.e., PTT) of the VM (66.6%, *p* < 0.01) compared to a control condition (10 s post iMVC), while no significant changes occurred in any H-reflex parameters (*p* > 0.05).

#### 3.4.5. The EMG H_max_/M_max_ Ratio and PAP/PAPE

One RCT investigated how the EMG H_max_/M_max_ ratio was related to the PAP/PAPE response [48]. This study found that the PTT acutely increased in the VM (6.7% to 66.6%, *p* < 0.05) compared to the control (10 s to 18 min post iMVC), followed by a greater EMG H_max_/M_max_ ratio (23.2% to 42%, *p* < 0.01), 5–11 min post the iMVC. In addition, when the twitch force (evoked by H_max_ stimulation) was expressed as a percentage of PTT, the twitch force was significantly higher (70.3% to 74.2%, *p* < 0.05) than the control (5–9 min post the iMVC). Noteworthily, the greatest EMG H_max_/M_max_ value (42%, *p* < 0.01) and the highest relative twitch force percentage (74.2%, *p* < 0.01) were both observed 5 min post the iMVC, although the highest mean value for the PTT occurred 10 s post iMVC. Still, this PAP response and the corresponding higher EMG H_max_/M_max_ ratio did not coincide with any acute improvements in iRFD or voluntary peak torque outcomes (*p* > 0.05). The H_max_ and M_max_ were obtained via motor nerve stimulation in this study.

## 4. Discussion

To our knowledge, this is the first systemic review that has evaluated if sEMG parameters are indicative of PAP/PAPE, in terms of twitch potentiation and voluntary performance. The two hypotheses were the following: (1) the PAP/PAPE magnitude will be positively related to the sEMG amplitude of the working muscle group (s), and (2) short-term increases in the sEMG frequency variables of the working muscle group (s) will also be positively related to PAP/PAPE. This review revealed that increases in sEMG amplitude (MAV and RMS) may only be indicative of PAP/PAPE in some muscles, in particular acute increases in voluntary performance (i.e., PAPE), and this relation seems to be influenced by several factors, including (1) the velocity and intensity of the CA, (2) how the EMG data were normalized and recorded, (3) the time point in which the EMG response was analyzed, and (4) accumulated neuromuscular fatigue. Additionally, this review revealed that an acute decrease in the MdF of the working muscles may (at least in some circumstances) be positively related to a PAP/PAPE response, when assessed in voluntary conditions (i.e., PAPE).

Furthermore, intriguingly, this review also found that the M-wave amplitude (together with a twitch torque outcome) may generally be indicative of a potentiated twitch response (i.e., PAP), whereas it remained unclear how it relates to PAPE. Similarly, changes in the EMG H_max_/M_max_ ratio were shown to contribute (temporally) to the evoked PAP response, while it was found to be unrelated to PAPE. However, interestingly, the H-reflex amplitude was shown to be neither indicative of PAP nor PAPE.

### 4.1. The sEMG Amplitude as an Indicative Measure of PAP/PAPE

Most studies in this review that reported a simultaneous increase in sEMG amplitude (in some muscles) and short-term improvements in voluntary muscular performance executed the CAs at maximal or near-maximal velocities, combined with moderate (60% of 1RM or at ∼70% of P_max_) [17,64] to high intensities (≥80% of 1 RM or ∼130% of P_max_, including DJs [15,17,18,34,43,66]). The higher sEMG amplitude (and the corresponding performance enhancement) occurred on average between 5 and 10 min post the CA [16,18,34,64], although one RCT [15] reported that individualized ICRIs may be needed to elicit any potentiating effect.

Nevertheless, many studies in this review used different normalization procedures for the EMG signal, although the muscles of interest were most commonly normalized to the RMS values obtained during a 1 RM attempt [34,64] or squat isometric lifts [18,66]. Further, all the performance outcomes that were subsequently evaluated were ballistic in nature, and predominately performed concentrically, including PPO [15,17,18], RFD [18], CMJ height [15,18], and MVP [34,64]. This suggests that assessing the sEMG amplitude may have some utility as an indicative measure of PAPE (at least in some muscles) during ballistic movements, focusing on maximum power development, while it generally may be a poor indicator of PAP outcomes (i.e., observing an acute increase in PTT or RTD_TW_), as one RCT in this review actually found that an acute increase in PTT coincided with a decreased MG RMS amplitude, and no myoelectrical changes in other calf muscles [63].

These findings support previous experimental studies inferring that the muscle potentiation effect is more prominent during peak muscle shortening speeds and high-speed concentric contractions, compared to isometric muscular activity [67,68]. However, it contradicts the importance of an increased expression of MRLC phosphorylation (i.e., the primary mechanism of PAP) for voluntary performance enhancement at high velocities, and instead signifies that other mechanisms, reflected by higher sEMG amplitude, may play a larger contributing role, at least when assessed during ballistic movements. Importantly, however, as it is well documented that the sEMG amplitude increases with higher muscle force but also with accumulated neuromuscular fatigue [30,32], it may be misleading to solely rely on acute increases in sEMG amplitude as an indicative measure of PAPE, without any direct performance assessment and/or evaluating other sEMG parameters.

### 4.2. The MdF as an Indicative Measure of PAP/PAPE

Based on the included studies in this review, an acute decrease in the MdF of the working muscles may (at least in some circumstances) reflect a PAP/PAPE response, when assessed in voluntary conditions (i.e., PAPE). While one good-quality RCT in this review reported that the MdF increased in the VL muscle following a DJ-CA protocol [43], these Mdf changes were not distinguishable from a control (a standard warmup) and occurred independent of any voluntary performance improvements. This may partially contradict the notion that increases in MdF are indicative of a higher proportion of type II fiber recruitment [57,58] or simply signify that PAPE is unrelated to an acute increased recruitment of type II fibers (at least in some circumstances). Alternatively, it implies that some other mechanism related to an acute decrease in the MdF may be indicative of PAPE.

Typically, EMG spectral variables (i.e., MdF and MPF) tend to decrease over time during fatiguing muscular contractions [30], and this has been attributed to a decline in muscle fiber velocity [33,69], via a reduction in intracellular pH [70,71]. Importantly, however, this reduction in MdF has repeatedly been found to coincide with an increased sEMG amplitude during fatiguing muscular contractions, as this has been proposed to reflect increased motor unit recruitment to maintain a constant muscle force [30,32]. Further, as muscle potentiation and fatigue normally coexist when performing any muscular activity, this may imply that a smaller drop in MdF (relative to the rise in sEMG amplitude) may be indicative of an active muscle that will be in a net potentiated or unpotentiated state. While a limitation with this review is that only two studies examined the occurrence of PAP/PAPE and MdF, this in part supports the findings from a good-quality RCT in this review.

In their study, Sun and Yang [16] demonstrated that the MdF of several lower body muscles (incl. VM, VL, RF, and BF) acutely decreased, while the RMS amplitude of the same muscles increased, after a BS-CA protocol combined with low intensity (i.e., 30% of 1RM) and BFRT at either 50%, 60%, or 70% AOP, respectively, compared to a control. However, short-term improvements in CMJ and SJ performance (i.e., PAPE) were only observed in the 50% and 60% AOP conditions, respectively. Further, the greatest performance improvements and lowest absolute drop in MdF (and rise in the RMS amplitude) of the VM, VL, RF, and BF occurred in the 50% AOP BFRT. However, intriguingly, the MdF in the 50% AOP BFRT was only lower (relative to the rise in the RMS amplitude) in the VM and RF muscle, respectively, compared to the 70% AOP BFRT and the control, while the MdF of the VL and BF was in contrast lower (relative to the RMS amplitude level) in the 70% AOP condition, compared to the 50% AOP and the control (see Section The RMS amplitude and PAP/PAPE and Section 3.4.2). Additionally, the GM RMS amplitude decreased in all AOP groups compared to baseline, with the lowest and highest decline observed in the 50% and 70% AOP groups, respectively. However, the MdF of the GM only increased in the 70% AOP condition.

This implies that a smaller drop in MdF (relative to the rise in the sEMG amplitude) of a muscle/muscle group may (or may not) be indicative of a PAPE response, as it seems to be highly dependent on the muscle (at least with BFRT). Based on previous research, this may be related to differences in muscle fiber type composition [72], especially the distribution of type II fibers in each muscle [73,74], and/or simply signify that the muscle activation pattern was more favorable in the 50% AOP condition for plyometric performance. For instance, in a kinematic and electromyographic study by Bobbert and van Ingen Schenau [75], it was reported that the RF and VM sEMG amplitude increased during the initial part of the push-off phase of a vertical jump in skilled jumpers, and this coincided with higher net moments around the knees. Concurrently, the BF sEMG amplitude also decreased during the push-off phase, which was explained by lower net moments around the hip joint. This suggests that an acute increase in the sEMG amplitude (relative to the drop in the MdF) in the RF and VM muscle, in addition to a lower BF sEMG amplitude, may only be indicative of PAPE for vertical jumping, while a different muscle activation pattern may be more favorable for other movements.

Further, while it is less certain how a reduction in the RMS amplitude and an increased MdF relates to fatigue within the neuromuscular system, a large decline in the MdF has consistently been shown to reflect neuromuscular fatigue in EMG research [29,31], which supports the findings in this review.

### 4.3. The M-Wave Amplitude as an Indicative Measure of PAP/PAPE

Six studies included in this review also investigated PAP/PAPE and changes in the evoked M-wave response (PtpA) obtained from either the VM, VL, SOL, MG, or LG muscle [10,35,43,48,62,63]. Intriguingly, all studies found that the evoked M-wave tended to remain the same, while significant improvements were observed in different PAP and PAPE outcomes. Noteworthily, the type of CA that was utilized, the muscle that was used for the M-wave assessment, and the performance outcome that was used for pre- and post-analyses did not significantly influence this relation. Intriguingly, however, one RCT [62] in this review observed a transient improvement in the evoked M-wave of the SOL, following three series of 5 s plantar flexion iMVCs. This M-wave enlargement or potentiation was noted immediately after the first and second iMVC, respectively, and 2–30 s post the last iMVC. However, it rapidly dropped to non-significant values at 90 s post the last iMVC. Similarly, the same study found that the PTT in the SOL was significantly higher at the second iMVC and 2–90 s post the last iMVC, but the values became non-significant at 150 s post the last iMVC. This infers that the action potentials that travel along the t-tubules to the muscle [10] may partially contribute to the initial development of PAP, suggesting that a transient rise in the evoked M-wave PtpA could be indicative of PAP. This short-lived M-wave potentiation (≤1 min) has been noted in previous studies [7,48] and has been attributed to the mechanism in the fiber membrane’s Na+-k+ active transport [7,76], but has also simply been suggested to be an artifact via subtle movements of the electrodes [48]. Regardless, collectively, the M-wave PtpA may generally be indicative of PAP (assuming it is combined with a twitch torque outcome), as it has consistently been reported to be unaffected by various CA protocols, and it has repeatedly been used to control for changes in neuromuscular propagation that could influence the potentiated twitch response (i.e., PAP) [10,40,41].

In contrast, whereas PAP tends to be highest immediately post an iMVC-CA protocol, and drop exponentially over time and sequentially disappear at 10–18 min post the CA [10,48,62,63], PAPE has generally been found to peak 5–10 min post the CA in most studies [6,16,18,34,64]. Therefore, any enhancement that the mechanisms related to the M-wave enlargement would temporally have on voluntary muscular performance would most likely be impaired by residual neuromuscular fatigue. This premise is supported by an experimental study by Hicks and colleagues [77], where the researchers noted that the M-wave values (area and PtpA) increased gradually during the first 2 min of the fatiguing protocol, and this coincided with a reduction in voluntary force. This suggests that assessment of the M-wave PtpA may mainly be applicable for PAP research (in addition to studying fatigue within the neuromuscular system), and that changes in the M-wave PtpA may generally not reflect PAPE. Still, one RCT included in this review actually reported that this M-wave enlargement (in the SOL) coincided with short-term increases in plantar flexor iRFD (i.e., PAPE), when it was analyzed in discrete units of time, compared to baseline (15–60 s post an iMVC-CA) [62]. This suggests that changes in neuromuscular propagation (e.g., sarcolemmal membrane excitability) may nevertheless partially contribute to the PAPE response, at least during brief explosive contractions. However, the same study [62] found that there were no significant differences in the iRFD when it was expressed as T_peak_ and RFD_avg_. Thus, how the M-wave PtpA relates to PAPE still needs to be explored, as the PAPE response seems to be strongly influenced by how the PAPE outcome is expressed and analyzed. In addition, as no studies in this review assessed the area and duration of the M-wave, and most studies included in this review only stimulated the motor nerve to obtain the M-wave [35,43,48,62,63], how these M-wave values and direct muscle stimulation relate to PAPE (and PAP) also remains to be elucidated.

### 4.4. The H-Reflex Amplitude as an Indicative Measure of PAP/PAPE

Two RCTs included in this review also investigated how PAP and PAPE were related to the evoked H-reflex response (PtpA) obtained from either the VM [48] or SOL [62] muscle. Both studies reported that the H-reflex remained the same, while significant improvements were observed in different PAP and PAPE outcomes. This implies that PAP and PAPE may both be unrelated to spinal excitability, signifying that changes in the H-reflex response may generally not be indicative of PAP nor PAPE. These findings are supported by a more recent study from Iglesias-Soler and coworkers [54]. In this study, the authors observed a short-term improvement in voluntary explosive plantar flexion force (i.e., PAPE) after an iMVC-CA protocol, but no observed differences in any H-reflex parameters (incl. amplitude and threshold). Based on previous work, this may be related to the fact that the H_max_ is elicited by submaximal nerve stimulation [44], and this has been found to primarily activate slow-twitch motor units [44,48]. In this regard, as MRLC phosphorylation has been observed to have less of an effect on slow-twitch skeletal muscles [6] and power-trained athletes tend to have lower amplitude of the H_max_ potential compared to their endurance-trained counterparts [44], it would further suggest that changes in the H-reflex response may be unrelated to PAP, but also should have minimal or no effect on acute increases in voluntary force production (i.e., PAPE).

### 4.5. The EMG H_max_/M_max_ Ratio as an Indicative Measure of PAP/PAPE

Lastly, one good-quality RCT [48] included in this review also examined how PAP and PAPE were related to the EMG H_max_/M_max_ ratio. In this study, Folland and co-workers [48] found that the maximal twitch force (i.e., PTT) was significantly greater 10 s to 18 min after the 10 s iMVC (i.e., a PAP response), compared to a control, and this was accompanied by a greater EMG H_max_/M_max_ ratio (5–11 min post iMVC). Additionally, the same study found that when twitch force at H_max_ was expressed as a percentage of PTT, the twitch force remained significantly higher than the control (5–9 min post the iMVC). Noteworthily, the greatest EMG H_max_/M_max_ value and the highest relative twitch force percentage was observed 5 min post the iMVC, although the highest mean value for the PTT occurred 10 s post iMVC.

This implies that the excitability of the motoneuron pool may temporally contribute to the evoked PAP response during twitch force assessment, signifying that the time point when the twitch response is analyzed impacts how twitch potentiation (i.e., PAP) relates to neurophysiological mechanisms. Based on the findings in this review, an increase in the EMG H_max_/M_max_ ratio may generally be indicative of a PAP response. However, the same study by Folland and colleagues [48] also found that a greater EMG H_max_/M_max_ ratio was not accompanied with short-term improvements in iRFD or voluntary peak torque, indicating that the EMG H_max_/M_max_ ratio may generally be a poor indicator of PAPE and thus may have limited sports applications, at least for strength-power athletes.

This supports experimental findings reporting that EMG H_max_/M_max_ ratios tend to be lower in athletes performing anaerobic compared to aerobic sports [44]. Intriguingly, power-trained athletes have also been found to have lower EMG H_max_/M_max_ ratios than sedentary subjects [78], which provides further evidence that the EMG H_max_/M_max_ ratio may generally be a poor indicator of PAPE. This highlights the inherent complexity with PAP/PAPE research. Still, there is a need for more high-quality RCTs and control trials that explore how various CAs, sEMG parameters, and twitch/performance outcomes interact, for us to construct better warmup guidelines to maximize muscular performance and minimize the incidence of musculoskeletal injuries. However, as the PAP/PAPE response has mainly been evaluated during dynamic movements, especially PAPE [6], it is currently unclear if CAs can also be utilized to induce short-term improvements in balance and stability of muscles, and if this can be measured effectively with sEMG. Further, in regard to injury prevention and musculoskeletal rehabilitation, this could have both sports and clinical applications.

### 4.6. Practical Applications

Based on the findings of this review, sports coaches and athletes interested in optimizing muscular power may be able to use the sEMG amplitude (i.e., MAV and RMS) to detect a PAPE response, when it is assessed during ballistic movements (e.g., during CMJs and BPTs). The most effective CA intensity was shown to be moderate (i.e., ~60% of 1 RM) to high intensity (i.e., ≥80% of 1 RM), executed at maximal or near-maximal velocity. The sEMG amplitude (and the corresponding PAPE effect) was found to be highest 5–10 min post the CA. Further, while a small decrease in the MdF was also shown to reflect a PAPE response, this review suggests that it may generally be a better tool for assessing excessive neuromuscular fatigue. Moreover, the M-wave amplitude (together with a twitch torque outcome) was shown to generally be indicative of PAP in this review, whereas changes in the EMG H_max_/M_max_ ratio were shown to contribute (temporally) to the evoked PAP response. A 5–10 s iMVC of the working muscle was the most common CA to elicit a potent PAP effect, followed by 2–10 s of recovery.

Interestingly, however, the PAP response remained detectable for up to 10–18 min post the CA (depending on the CA protocol). Having a greater understanding of the neurophysiological mechanisms that could directly enhance or impair the development of muscle force could be of great importance for researchers examining the underlying mechanism of muscle weakness. Thus, understanding how sEMG parameters relate to PAP (and PAPE) could also be of great value for healthcare professionals rehabilitating (or managing) patients with various neuromuscular disorders.

### 4.7. Limitations and Recommendations for Future Research

While some strengths with this review are that the PAP and PAPE outcomes were clearly defined and separated (Table 2), and the studies included had excellent quality on average (see Section 3.3 and Table 1), there are still several methodological limitations with the present study. This includes the small sample size in this review, and the lack of good-quality RCTs on this topic (in general). Additionally, it was found that several studies used different normalization procedures for the EMG signal and the subjects included in this review were restricted to healthy athletic populations. This, combined with the limited number of studies, may limit the generalizability of the findings. Hence, there is undoubtedly a need for higher-quality studies on this topic. Future studies should for instance use more standardized normalization protocols but also examine how several different sEMG parameters are related to various CA protocols to further improve our understanding of the underlying neurophysiology of PAP/PAPE. Moreover, how sEMG parameters are influenced by different muscle contraction modes (i.e., isometrics, concentrics, and eccentrics) and how this relates to the PAP/PAPE response should also be explored. Lastly, how the M-wave, obtained via direct muscle stimulation, and different M-values (e.g., area and duration) relate to PAP/PAPE also remains to be elucidated.

## 5. Conclusions

This review aimed to identify if sEMG parameters are indicative of post-activation potentiation (PAP)/PAPE, in terms of twitch potentiation and voluntary performance. The findings in this review showed that the M-wave amplitude (combined with a twitch torque outcome) may generally be indicative of PAP. The sEMG amplitudes (in some muscles) were found to be indicative of PAPE during ballistic, high-power output movements, while a small decrease in the MdF (in certain muscles) was shown to reflect a PAPE response, although it generally seems to be a better tool for controlling excessive neuromuscular fatigue. On the contrary, changes in the EMG H_max_/M_max_ ratio were found to contribute (temporally) to the evoked PAP response, while the H-reflex amplitude was shown to be neither indicative of PAP nor PAPE. This review provides preliminary findings suggesting that certain sEMG parameters could be indicative of PAP/PAPE. However, due to the limited number of good- to high-quality RCTs on this topic, further research is warranted to re-examine how different sEMG parameters are related to the PAP/PAPE phenomenon. Having a greater understanding of how sEMG parameters relate to PAP/PAPE may help us reduce the incidence of musculoskeletal injuries in athletes and the general population, by providing us with new tools to construct better warmup guidelines. Clinically, this may also be of great importance for healthcare professionals rehabilitating patients with muscle weakness, a condition affecting millions of older adults worldwide. This review therefore highlights the potential value of understanding which parameters of sEMG can be used to detect PAP/PAPE.

## Figures and Tables

**Table 1:** PEDro Rating of the included studies.

Studies				Criteria					
	Item 1	Item 2	Item 3	Item 4	Item 8	Item 9	Item 10	Item 11	Total
Barnes et al. [61] (2017)	Yes	1	1	1	1	1	1	1	**7**
Scott et al. [15] (2018)	Yes	1	0	0	1	1	1	1	**5**
Mina et al. [18] (2018)	Yes	1	0	1	1	1	1	1	**6**
Johnson et al. [10] (2019)	Yes	1	0	1	1	1	1	0	**5**
Seitz et al. [35] (2015)	Yes	1	0	1	1	1	1	1	**6**
Hodgson et al. [62] (2008)	Yes	1	0	1	1	1	1	1	**6**
Miyamoto et al. [63] (2011)	Yes	1	0	1	1	1	1	1	**5**
Sotiropoulos et al. [17] (2014)	Yes	1	0	1	1	1	1	1	**6**
Tsoukos et al. [64] (2019)	Yes	1	0	1	1	1	1	1	**6**
Folland et al. [48] (2008)	Yes	1	0	1	0	1	1	1	**5**
Mina et al. [65] (2014)	Yes	1	0	1	1	1	1	1	**6**
Sun & Yang [16] (2023)	Yes	1	0	1	1	1	1	1	**6**
Zagatto et al. [43] (2022)	Yes	1	0	0	0	1	1	1	**4**
Mina et al. [66] (2016)	Yes	1	0	1	1	1	1	1	**6**
Tsoukos et al. [34] (2021)	Yes	1	0	1	1	1	1	1	**6**

Item 1 = eligibility criteria were specified; item 2 = subjects were randomly allocated to groups; item 3 = allocation was concealed; item 4 = the groups were similar at baseline regarding the most important prognostic indicators; item 8 = measures of one key outcome were obtained from 85% of subjects initially allocated to groups; item 9 = all subjects for whom outcome measures were available received the treatment or control condition as allocated or, where this was not the case, data for at least one key outcome were analyzed by “intention to treat”; item 10 = the results of between-group statistical comparisons were reported for at least one key outcome; item 11 = the study provided both point measures and measures of variability for at least one key outcome. 1 = clearly described and presented in detail; 0 = absent, insufficiently described, or unclear.

**Table 2:** The characteristics of the included studies.

Study (Authors)	N and Sex of Subjects	Mean Age in Years (±SD)	Training Status	CA Protocol	sEMG Parameter (Outcome)	Twitch/Performance Measures (PAP and PAPE Outcomes)	Main Findings
Barnes et al. [61] (2017)	9 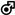	(23.7 ± 3.8)	Active subjects; ≥3 years of RT experience.	Six Warm Up Modalities: HPS, cycling, WBV, Cycle + HPS, WBV + HPS and a CON.	**sEMG amplitude** (RMS) of the VL, BF and GM muscles.	**PAPE**—PPO during the high pull exercise.	**No significant changes** in the **sEMG amplitude** of any muscle. ↑ **PPO** in the HPS and WBW + HPS condition respectively, compared to the CON. **No significant changes** in the **PPO** in the cycling, WBV, Cycle + HPS, WBV + HPS.
Scott el al. [15] (2018)	20 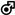	(22.35 ± 2.68)	Amateur rugby league players.	HBD or BS at 70% of 1RM, combined with accommodating resistance (varied from 0 to 23% 1RM across the ROM). CON (no CA).	**sEMG amplitude** (MAV) of the VL, RF, TA & MG.	**PAPE**—CMJ Performance (Height and PPO)	↑ **VL, RF, TA** and **MG sEMG amplitude**, compared to baseline CMJs (when ICRIs were individualized). ↑ **CMJ Height** compared to baseline CMJs, 30 s post the HBD or BS protocol. ↑ **PPO** and **CMJ Height** compared to baseline CMJs when ICRIs were individualized, post the HBD or BS protocol. **No significant changes** in the **EMG amplitude** of any muscle, when investigated at prescribed ICRIs.
Mina et al. [18] (2018)	15 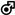	(21.7 ± 1.1)	Active subjects; ≥5 years of RT experience.	BS at 85% of 1RM with FWR (CON) and VR (35% of the total load)	Concentric peak and mean **sEMG amplitude** (RMS) of the VL, VM and GM. Eccentric peak and mean **sEMG amplitude** (RMS) of the VL, VM and GM	**PAPE**—CMJ Performance (Height, PPO and RFD)	↑ **Mean concentric VL sEMG amplitude** in the VR condition, compared to baseline and the CON. **No significant changes** in the **VM** and **GM sEMG amplitude.** ↑ **PPO**, **RFD** and **CMJ height** in the VR condition.
Johnson et al. [10] (2019)	20 (12 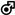 , 8 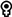 )	(22.1 ± 0.60)	Athletic population.	DJs and Low-Pace Walking (CON)	**M-wave** (PtpA) of the VM muscle.	**PAP**—PTT and RTD_TW_ in the VM	**No significant changes** in the **M-wave PtpA** of the **VM**. ↑ **PTT** and **RTD_TW_** in the **VM** immediately post the DJ protocol, compared to CON.
Seitz et al. [35] (2015)	17 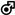	(25.4 ± 3.9)	Active subjects; ≥6 months of RT experience.	Two IKKEs at CA60/4, CA180/12, CA300/20, CA180/4 (CON) and CA300/4	**sEMG amplitude** (RMS) of the VM, RF and VL muscles. **M-wave** (PtpA) of the VL muscle.	**PAP**—PTT in the VL **PAPE**—VPT during IKKEs	**No significant changes** in the **sEMG amplitude** of any muscle. **No significant changes** in the **M-wave PtpA** of the **VL**. ↑ **VPT** from 4 to 7 min-post-CA during CA60/4, CA180/12, and CA300/20, compared to baseline. ↑ **PTT** from 1 to 4 min post-CA for CA60/4, CA180/12, and CA300/20, compared to baseline values.
Hodgson et al. [62] (2008)	13 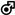	(23.5 ± 2.4)	Active subjects; ≥2 years of RT experience and/or playing sports at interuniversity level or above.	Plantar flexion iMVC plus explosive plantar flexions. CON (only plantar flexion iMVC)	**H-reflex** (PtpA) of the SOL muscle (via stimulating the posterior tibial nerve). **M-wave** (PtpA) the SOL muscle (via stimulating the posterior tibial nerve).	**PAP**—PTT in the SOL **PAPE**—iRFD during explosive plantar flexions (Measured as T_peak_, RFD_avg_ and discrete time intervals)	**No significant changes** in the **H-reflex PtpA** of the **SOL**.↑ **M-wave PtpA** of the **SOL**, immediately after the 1st to 2nd iMVC respectively, and 2–30 s post the 3rd iMVC in the CA condition, compared to the baseline. ↑ **PTT** in the **SOL**, immediately after the 2nd iMVC and 2–90 s post the 3rd iMVC in the CA condition, compared to the baseline. ↑ **iRFD** during explosive plantar flexions, when measured in discrete time intervals in the CA condition, compared to the baseline. **No significant changes** in the plantar flexor **RFD_avg_** and **T_peak_**.
Miyamoto et al. [63] (2011)	9 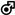	(26.7 ± 4.4)	Active subjects; ≥1 years of RT experience.	Three IKPFs * at 180°/s plus 6 s iMVC (EXP). Three IKPFs * at 180°/s (No iMVC, CON)	**sEMG amplitude** (RMS) of the LG, MG, SOL and TA muscles. **M-wave** (PtpA) of the LG, MG, and SOL muscle.	**PAP**—PTT in the LG, MG and SOL respectively. **PAPE**—VPT during IKPFs.	↓ **MG sEMG amplitude** immediately after the iMVC, compared to baseline levels and the CON. **No significant changes** in the **sEMG amplitude** of the LG, SOL and TA. **No significant changes** in **M-wave PtpA** of any muscle. ↑ **PTT** in the **LG, MG** and **SOL**, from immediately after up to 5 min post iMVC, compared to the CON.↑ **VPT** from 1 to 3 min-post-CA during IKPFs at 180°/s, compared to baseline levels and the CON.
Sotiropoulos et al. [17] (2014)	12 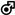	(20.1 ± 3.3)	Volleyball players.	Loaded SJs at either P_max,_ 70% of P_max_ or 130% of P_max_. No SJ (Con).	Concentric mean **sEMG amplitude** (RMS) of the * QF and BF muscle. * The QF EMG activity was calculated from the mean activity of the RF, VL and VM.	**PAPE**—RJ Performance (Height and PPO)	↑ **Mean concentric QF sEMG amplitude** in 130%P_max_ and 70%P_max_ condition from 1–10 min respectively, post the CA protocols, compared to the control. ↑ **PPO** in 70%P_max_ and 130%P_max_, from 5–7 min, and the 5th min respectively post the CA protocols, compared to the control. **No significant changes** in the **RJ height**.
Tsoukos et al. [64] (2019)	11 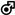	(26.4 ± 6.5)	Healthy active subjects with athletic backgrounds: ≥3 years of RT experience.	BP exercise at 40% or 60% of 1 RM, with C90 & C70. Four EXPs: (a) BP at 40% of 1 RM with C90, (b) BP at 40% of 1 RM with C70, (c) BP at 60% of 1 RM with C90, and (d) BP at 60% of 1 RM with C70. CON (performed only the BPT).	**sEMG amplitude** (RMS) of the PM and TB during MPV (at 0.75, 2, 4, 6, 8, 10 and 12 min).	**PAPE**—mean concentric MPV and PV during the BPT (at 0.75, 2, 4, 6, 8, 10 and 12 min)	↑ **TB sEMG amplitude** during **MVP** in the C90 condition with 60% of 1 RM, during the highest achieved **MVP** compared to baseline, the C70 condition with 40% of 1 RM and the CON. **No significant changes** in the **PM EMG amplitude** in any condition or time course of recovery. ↑ **Mean concentric MVP** in the C90 condition with 60% of 1 RM, from 4–12 min of recovery, compared to baseline and the CON. ↑ **Mean concentric MVP** in the C90 condition with 60% of 1 RM, from 0.75–10 min of recovery, compared to the C70 condition with 60% of 1 RM, and C70 condition 40% of 1RM respectively. ↑ **Mean concentric PV** in the C90 condition with 60% of 1 RM, from 4–12 min of recovery, compared to baseline, the C70 condition with 60% of 1 RM and the CON.
Folland et al. [48] (2008).	8 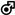	(25.0 ± 3.0)	Healthy recreationally active subjects.	10 s iMVC (knee extension) at 100° knee flexion (EXP) Controlled rest (CON)	**H-reflex** (PtpA) of the VM muscle (to obtain H_max_). **M-wave** (PtpA) the VM muscle (to obtain M_max_). **EMG H_max_/M_max_ ratio** (measured for 18 min, after a period of rest (CON) and 10 s iMVC).	**PAP**—PTT and relative TF at H_max_ in the VM respectively. **PAPE**—iRFD during knee extension and VPT (IKKEs at 240°·s^−1^)	**No significant changes** in the **M_max_** response of the **VM** between the EXP and CON. ↑ **PTT** in the **VM**, from 10 s to 18 min post iMVC compared to the CON. ↑ **Relative TF at Hmax** in the **VM**, from 5–9 min post iMVC compared to the CON. ↑ **EMG H_max_/M_max_ ratio** of the **VM**, from 3–11 min post iMVC compared to the CON. **No significant changes** in **iRFD** or **VPT**, compared to the CON.
Mina et al. [65] (2014).	16 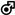	(26.0 ± 7.8)	Active subjects; ≥3 years of RT experience.	BS at 85% of 1RM with FWR (CON) and VR (35% of the total load)	Concentric peak and mean **sEMG amplitude** (RMS) of the RF, VL and ST Eccentric peak and mean **sEMG amplitude** (RMS) of the RF, VL and ST	**PAPE**—1RM BS Performance (Mean Load)	**No significant changes** in the sEMG amplitude of any muscle. ↑ **1RM BS performance** was observed in the VR condition compared to the CON.
Sun & Yang. [16] (2023).	12 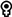	(18.34 ± 1.88)	Elite Soccer Players; >3 years of RT experience.	Semi-Squats, with no-BRFT (CON), with BRFT at 50% of AOP, 60% of AOP and 70% of AOP (In all protocols, 30% of 1RM and 4 sets/75 reps (30–15–15–15) with seconds interval time was used)	**sEMG amplitude** (RMS) of the, RF, VM, VL, BF, GM and Gmeds muscles. **MdF** of the, RF, VM, VL, BF, GM and Gmeds muscles.	**PAPE**—CMJ and SJ performance respectively (Height, PPO and RFD)	↑ **RF, VM, VL and BF sEMG amplitude** during the BRFT condition at 50%, 60% and 70% of AOP, compared to baseline and the CON. ↓ **GM sEMG amplitude** during BRFT condition at 50%, 60% and 70% of AOP, compared to baseline. ↓ **MdF** of the **RF, VM, VL, BF** during the BRFT condition at 50%, 60% and 70% of AOP, compared to baseline and the CON. ↑ **MdF** of the **GM** during the BRFT condition at 70% of AOP, compared to baseline. ↓ **MdF** of the **Gmeds** during the BRFT condition at 50%, 60% and 70% of AOP, compared to the CON. ↑ **CMJ-height**, **SJ-height**, PPO* and RFD* at 50% and 60% of AOP, with 5 min and 10 min resting compared to baseline values. ↑ **CMJ-height**, **SJ-height**, PPO* and RFD* at 50% of AOP, at 5 min resting compared to the CON. * During CMJ and SJ respectively.
Zagatto et al. [43] (2022)	10 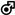	(17.5 ± 1.2)	Basketball players.	DJs, Sled Towing vs. No exercise (CON).	**sEMG amplitude** (RMS) of the MG, RF, VL & BF muscles. **MdF** of the MG, RF, VL & BF muscles. **M-wave** (PtpA) of the VL muscle.	**PAPE**—RSA outcomes (best time, mean time, total time, and slowest time)	↑ **BF sEMG amplitude** in the DJ condition, compared to the CON during the RSA testing. ↓ **RF sEMG amplitude** during the 4th and 10th compared to the 1st sprint in DJ condition. ↑ **MdF** of the **VL** in all conditions compared to baseline. **No significant changes** in **M-wave PtpA** of the **VL**. ↑ **RSA mean time**, **total time**, **slowest time** 4 min post the DJ protocol, compared to CON. **No significant changes** in any **RSA outcome** in the heavy sled towing condition.
Mina et al. [66] (2016).	16 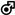	(26.0 ± 7.8)	Active subjects; ≥3 years of RT experience.	BS at 85% of 1RM with FWR (CON) and CLR (35% of the total load)	Concentric peak **and** mean **sEMG amplitude** (RMS) of the RF, VL, VM and ST. Eccentric peak and mean **sEMG amplitude** (RMS) of the RF, VL, VM and ST	**PAPE**—1RM BS Performance (Mean Load)	↑ **Eccentric Mean QF sEMG amplitude** in the CLR condition, compared to the CON. ↑ **1RM BS performance** was observed in the CLR condition compared to the CON.
Tsoukos et al. [34] (2021).	11 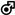	(26.5 ± 6.5)	Healthy active subjects with athletic backgrounds: ≥3 years of RT experience.	Heavy loaded BP at 80% of 1RM with C90 & C70. CON (performed only the BPT).	**sEMG amplitude** (RMS) of the PM and TB during MPV (at 0.75, 2, 4, 6, 8, 10 and 12 min)	**PAPE**—mean concentric MPV and PV during the BPT (at 0.75, 2, 4, 6, 8, 10 and 12 min)	↑ **PM sEMG amplitude** during **MVP** in the C90 condition, following 10 min of recovery, compared to baseline, the C70 condition and the CON. ↑ **PM sEMG amplitude** in the C90 condition, during the highest achieved **MVP** compared to baseline, the C70 condition and the CON. **No significant changes** in the **TB EMG amplitude** in any condition or time course of recovery. ↑ **Mean concentric MVP** in the C90 condition, from 4–12 min of recovery, compared to baseline. ↑ **Mean concentric MVP** in the C70 condition, from 10–12 min of recovery, compared to baseline. ↑ **Mean concentric PV** in the C90 condition, from 8–12 min of recovery, compared to baseline and the CON.

**1RM** = One Repetition Maximum; **AOP** = Arterial Occlusion Pressure; **BF** = Biceps Femoris; **BP** = Bench Press; **BPT** = Bench Press Throw; **BRFT** = Blood Flow Restriction Training; **BS** = Back Squat; C70 = Mean velocity of reps dropped to 70% of the highest attained; **C90** = Mean velocity of reps dropped to 90% of the highest attained; **CA** = Conditioning Activity; **CA60/4** = 4 reps at 60°·s^−1^; **CA180/12** = 12 reps at 180°·s^−1^; **CA300/20** = 20 reps at 300°·s^−1^; **CA180/4** = 4 reps at 180°·s^−1^; **CA300/4** = 4 reps at 180°·s^−1^; **CLR** = Chain-loaded resistance; **CMJ** = Countermovement jump; **CON** = Control Condition; **DJ** = Drop Jump; **EXP** = Experimental Trial; **FWR** = Free-weight resistance; **GM** = Gluteus Maximus; **Gmeds** = Gluteus Medius; **HBD** = Hex bar deadlift; **HPS** = High-Pull Specific; **ICRIs** = Intra Complex Recovery Intervals; **IKKE** = Isokinetic Knee Extension; **IKPF**= Isokinetic Plantar Flexion; **iMVC** = Isometric Maximum Voluntary Contraction; **iRFD** = Isometric Rate of Force Development; **LG** = Lateral Gastrocnemius; **MAV** = Mean Absolute Value; **MdF** = Median Frequency; **MG** = Medial Gastrocnemius; **MPV** = Mean Propulsive Velocity; **N** = Sample Size; **PAP**; Post-activation potentiation; **PAPE**; Post-activation performance enhancement; **PM** = Pectoralis Major; **P_max_** = Maximized mechanical-power output; **PPO** = Peak Power Output; **PtpA** = Peak-to-peak amplitude; **PTT** = Peak Twitch Torque; **PV** = Peak Velocity; **Reps** = Repetitions; **RF** = Rectus Femoris; **RFD** = Rate of Force Development; **RFD_avg_** = Average RFD; **RJ** =Repeated Jump; **RMS** = Root Mean Square; **ROM** = Range of Motion; **RSA** = Repeated sprint ability; **RT** = Resistance Training; Development; **RTD_TW_**= Twitch Rate of Torque Development; **SD** = Standard Deviation; **SJ** = Squat Jump; **SOL** = Soleus; **sEMG** = Surface electromyography; **ST** = Semitendinosus; **TA** = Tibialis Anterior; **TB** = Triceps Brachii; **TF** = Twitch Force; **T_peak_** = Time to peak force; **VL** = Vastus Lateralis; **VM** = Vastus Medialis; **VPT** = Voluntary Peak Torque; **VR** = Variable Resistance; **WBV** = Whole Body Vibration; **QF**, Quadriceps femoris; ↑ indicates significant increase; ↓ indicates significant decrease 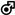 indicates male; 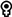 indicates female. * During the concentric phase.

## Data Availability

No new data were created or analyzed in this study. Data sharing is not applicable to this review.

## Supplementary Materials

The following supporting information can be downloaded at: www.mdpi.com/review/10.3390/xxx/s1, Table S1: Review matrix of included studies.
